# Dynamic interplay between RNA *N*^6^-methyladenosine modification and porcine reproductive and respiratory syndrome virus infection

**DOI:** 10.1186/s13567-025-01495-y

**Published:** 2025-03-22

**Authors:** Zi-Han Wang, Jing Li, Sai-Ya Ma, Meng-Xuan Liu, Yu-Fei Zhan, Feng Jin, Bing-Xin Liu, Wenjing Wang, Mei He, Yu-Chuan Yang, Yandong Tang, Peng Wang, Wuchao Zhang, Jie Tong

**Affiliations:** 1 College of Life Sciences, School of Life Sciences and Green Development, Hebei University, Baoding, 071002 China; 2 College of Veterinary Medicine, Hebei Agriculture University, Baoding, 071001 China; 3 State Key Laboratory for Animal Disease Control and Prevention, Harbin Veterinary Research Institute, Chinese Academy of Agricultural Sciences, Harbin, 045100 China; 4 Hebei Provincial Hospital of Traditional Chinese Medicine, Shijiazhuang, 050000 China; 5 Neural Academy of Traditional Chinese Medicine, Hebei University of Chinese Medicine, Shijiazhuang, 050000 China

**Keywords:** *N*^*6*^-methyladenosine (m^6^a), porcine reproductive and respiratory syndrome virus (PRRSV), virus replication, m^6^A RNA immunoprecipitation sequencing (m^6^A-seq), p38/MAPK signalling pathway

## Abstract

*N*^*6*^-methyladenosine (m^6^A) has attracted significant attention for its role in regulating the complex interaction between viruses and host cells. Porcine reproductive and respiratory syndrome virus (PRRSV) is a significant pathogen affecting swine health worldwide. Here, we first identified seven m^6^A-enriched peaks in PRRSV genomic RNA by m^6^A RNA immunoprecipitation sequencing (m^6^A-seq). Moreover, functional analyses revealed a positive correlation between the m^6^A modification level and PRRSV replication. Treatment with the universal methylation inhibitor 3-deazaadenosine (3-DAA) effectively suppressed PRRSV replication in a dose-dependent manner. Furthermore, m^6^A-seq was also used to determine the m^6^A landscape of the transcriptome in PAMs infected with pandemic or highly pathogenic PRRSV strains. Among the 4677 transcripts exhibiting altered m^6^A modification levels, the MAPK14 gene and the p38/MAPK signalling pathway emerged as preliminary targets of m^6^A-mediated epigenetic regulation during PRRSV infection. These findings provide new insights into the epigenetic mechanisms underlying PRRSV infection and may facilitate the development of anti-PRRSV therapeutics.

**Supplementary Information:**

The online version contains supplementary material available at 10.1186/s13567-025-01495-y.

## Introduction, methods and results

The role of RNA modifications as critical regulators of gene expression has been explored in the context of viral infections over the past several years [1–3]. Among these modifications, *N*^6^-methyladenosine (m^6^A) has attracted significant attention for its role in various biological processes, including RNA stability, translation, and the immune response [4, 5]. m^6^A is the most prevalent internal modification in eukaryotic messenger RNA (mRNA), and its dynamic nature allows rapid alterations in mRNAs in response to intra- and extracellular insults [6–8]. The importance of m^6^A extends beyond cellular RNA to include viral RNA, as many viruses exploit this modification to increase their replication and evade host immune defenses [9]. m^6^A may regulate the antiviral immune response via several potential mechanisms. For example, m^6^A modifications can influence the expression of key immune-related genes by dynamically regulating mRNA stability, splicing, translation, and degradation. m^6^A modifications also play critical roles in antiviral responses by modulating the stability and translation of pattern recognition receptor (PRR) transcripts and downstream signalling components (e.g., RIG-I and IFN-β). Moreover, viruses can exploit m^6^A modifications to evade immune responses, as viral RNA often acquires or manipulates m^6^A marks to increase replication or suppress host defenses [10].

Porcine reproductive and respiratory syndrome virus (PRRSV) is a significant pathogen affecting swine health worldwide, leading to considerable economic losses in the pig industry [11]. Understanding the mechanisms of PRRSV infection is essential for developing effective control strategies. The emergence of PRRSV was first documented in the United States and Europe in the late 1980s [12]. The situation intensified in 2006 with the epidemic of a highly pathogenic variant of PRRSV (HP-PRRSV) in China, which led to more severe outbreaks characterized by higher morbidity and mortality rates [13–15]. PRRSV NADC30-like strains first emerged in China in 2013 [16]. These strains share genetic similarity with the NADC30 strain isolated in the United States, which has a 131-amino acid deletion within the hypervariable region of the nsp2 protein [17]. Lin et al. [18] and Gong et al. [19] suggested that m^6^A modifications are important for PRRSV infection and host antiviral immune regulation, but neither study directly validated the functional roles of specific m^6^A sites on PRRSV RNA or host immune-related transcripts. A comprehensive view of the networks regulated by m^6^A during PRRSV infection is lacking. Our study employed functional analysis to identify the exact roles of m^6^A modification during PRRSV infection and identify specific m^6^A sites on PRRSV genomic RNA. MeRIP-seq also provides a comprehensive overview of the gene networks in host cells regulated by m^6^A during PRRSV infection. These findings may provide new insights into the epigenetic mechanisms underlying viral infection and identify potential antiviral targets.

### Mapping the m^6^A peaks in PRRSV genomic RNA

**Figure 1:**
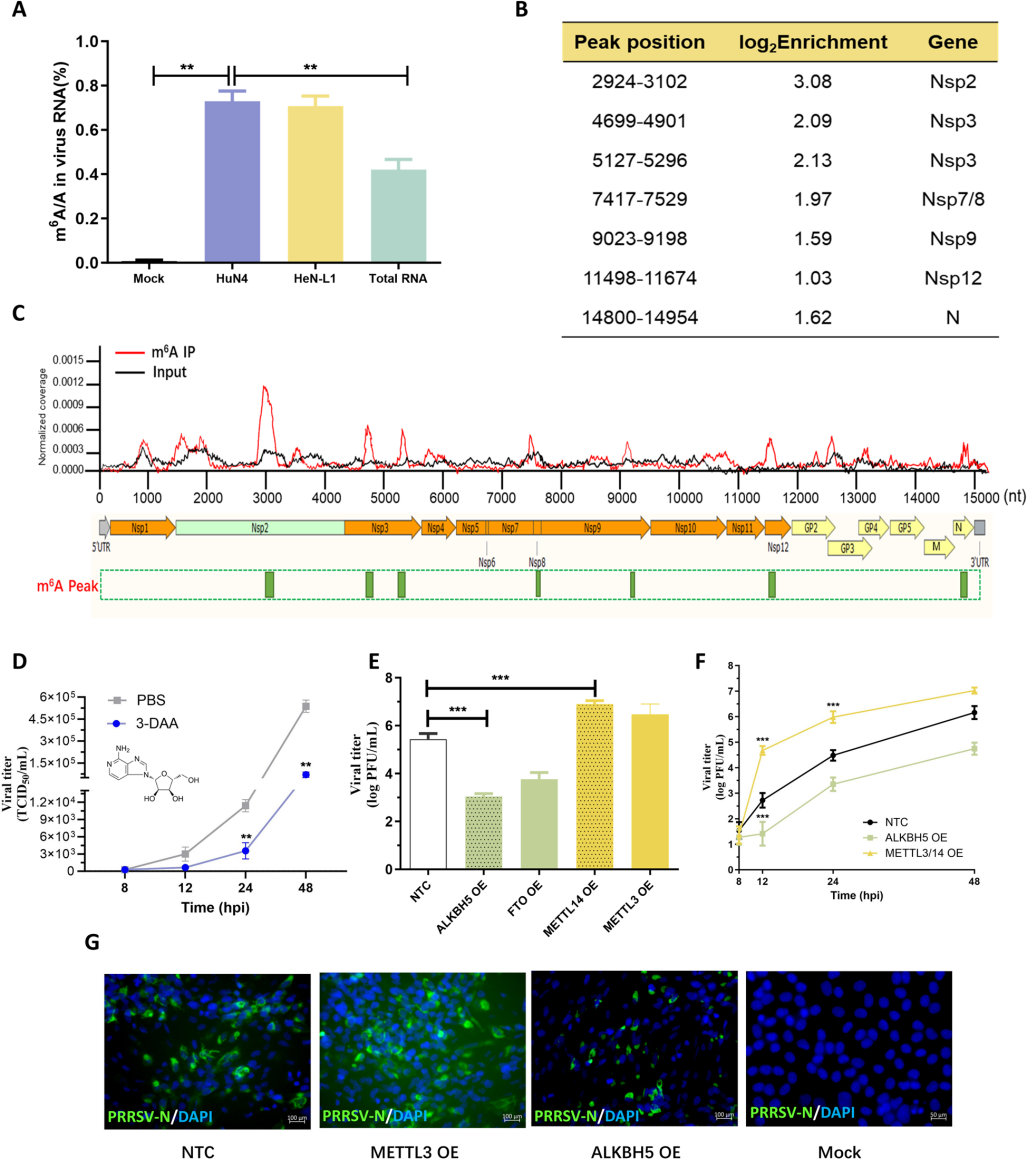
**Mapping the m**^6^**A peaks in the PRRSV genome and analysing the correlation between m**^6^**A modification and PRRSV replication.** **A** Elisa-based methods were used to determine the ratio of m^6^A/A in the mRNAs of PRRSV-infected or uninfected PAM cells. **B** and **C** Mapping the m^6^A-enriched peaks in PRRSV genomic RNA. The stringent peak calling method (FDR < 0.01) was applied to determine the statistically significant peaks (*p* values < 1E-10). **D** Determination of the viral titre via the TCID_50_ assay. Mrac145 cells were treated with 3-DAA or PBS prior to PRRSV infection. **E** and (**F**) Overexpression of m^6^A enzymes in PRRSV-infected Marc145 cells. The viral titre was determined via plaque assay. **G** IFA detection of the PRRSV N protein (green) in m^6^A enzyme-overexpressing Marc145 cells. DAPI was used to stain the cell nucleus. Statistical relevance was determined with an unpaired Student’s *t*‐test, **p < 0.001.

In our study, we utilized the highly pathogenic HuN4 strain and pandemic NADC-30, similar to the HeN-L1 strain (Additional file 1). We first employed an ELISA-based approach to assess the presence of m^6^A modifications in PRRSV genomic RNA. Porcine alveolar macrophages (PAMs) were infected with either the PRRSV HuN4 strain or the PRRSV NADC30-like strain, and after 48 h, the cells were subjected to three freeze‒thaw cycles. The supernatants were collected by centrifugation, followed by ultrafiltration to concentrate the viral particles, from which the viral RNA was extracted and fragmented into 80–200-nt fragments. The presence of m^6^A was then measured as a percentage of all adenosines in the viral RNA sample using ELISA. As shown in Figure 1A, the percentage of viral RNA with m^6^A modification was approximately 0.6–0.8%, which was twice as high as the 0.3–0.4% observed in total cellular RNA (Figure 1A). Figure 1A highlights the baseline differences in global m^6^A levels between porcine alveolar macrophages (PAMs) infected with the PRRSV HuN4 strain and those infected with the NADC30-like strain. Using m^6^A-specific ELISA, we detected significantly greater m^6^A enrichment in both HuN4- and HeN-L1-infected cells than in uninfected cells, indicating the common upregulation of m^6^A modification during infection with diverse PRRSV strains. These findings provide the foundation for investigating the distinct regulatory roles of m^6^A in the host–virus interplay and highlight the importance of further exploration of the pathways modulated by m^6^A. To further map the m^6^A-modified regions within the viral genome, MeRIP-seq was conducted by DIATRE Biotechnology, Shanghai, China. The experimental flow of MeRIP-seq is shown in Additional files 1 and 2. Briefly, PAMs were infected with the HeN-L1 strain, and at 48 hours post-infection (hpi), the cells were subjected to three freeze‒thaw cycles, followed by centrifugation and ultrafiltration to concentrate the viral particles. The extracted viral RNA was fragmented and immunoprecipitated with a m^6^A-specific antibody. Reverse transcription and subsequent deep sequencing of the RNA‒antibody complexes were applied to identify the m^6^A-modified region in PRRSV genomic RNA. The stringent peak calling method (FDR < 0.01) was applied to determine the statistically significant peaks (*p* values < 1E-10). A correlation test between three biological replicates of viral RNA revealed a high correlation (0.996), which confirmed the replicability of the results. Seven m^6^A-enriched regions were revealed in the viral genome, with one located in the N protein-coding region and the others distributed across nonstructural protein-coding regions. Notably, the Nsp2-encoding region contained the highest m^6^A peak, the length of which was approximately 178 nt (Figures 1B and C).

### Positive correlation between m^6^A modification levels and PRRSV replication

Previous studies have shown that the PRRSV genome does not encode kinases that mediate m^6^A modification. Therefore, we first interfered with the expression of endogenous m^6^A-related enzymes [20, 21]. Marc145 cells were seeded in a six-well plate, and when the cell confluence reached 60%, 3-deazaadenosine (3-DAA, 25 µM) was used to inhibit the methylation of adenosine [22]. Twenty-four hours after 3-DAA treatment, the cells were infected with HeN-L1 (MOI = 0.1). Eight to 48 h after virus infection, the cells were subjected to repeated freeze‒thaw cycles, and the virus in the supernatant was collected for the TCID_50_ assay to determine the virus titre. As shown in Figure 1D, compared with PBS treatment, 3-DAA treatment significantly decreased the virus titre from 24 to 48 h. To investigate the specific effects of m^6^A enzymes on PRRSV replication, Marc145 cells overexpressing (OE) m^6^A writers or erasers were used. As shown in Figure 1E, when ALKBH5 or FTO was overexpressed, the virus titre was significantly reduced at 12 hpi, whereas in cells overexpressing METTL14 or METTL3, the virus titre increased to approximately 10^7.0^ PFU/mL. To further validate the effects of m^6^A enzymes on viral replication kinetics, the viruses were collected at 8, 12, 24, and 48 hpi. As shown in Figure 1F, the replication of PRRSV began to be affected by manipulation of endogenous m^6^A enzymes after 8 hpi, and this effect lasted until 48 hpi. To detect the effects of m^6^A modification on the expression of virus-encoded proteins, Marc145 cells were seeded in 24-well plates, and m^6^A enzymes were overexpressed via lentiviral vectors. Eight hours post-transfection, the cells were infected with HeN-L1 (MOI = 0.1). At 24 hpi, the cells were fixed, and an indirect immunofluorescence assay (IFA) was used to detect the expression of the PRRSV N protein. As shown in Figure 1G, compared with the backbone vector, transient overexpression of METTL3 increased virus replication, whereas ALKBH5 expression decreased virus replication. These results preliminarily indicate that m^6^A modification may positively regulate PRRSV replication.

### The m^6^A landscape of the transcriptome altered by PRRSV infection

**Figure 2:**
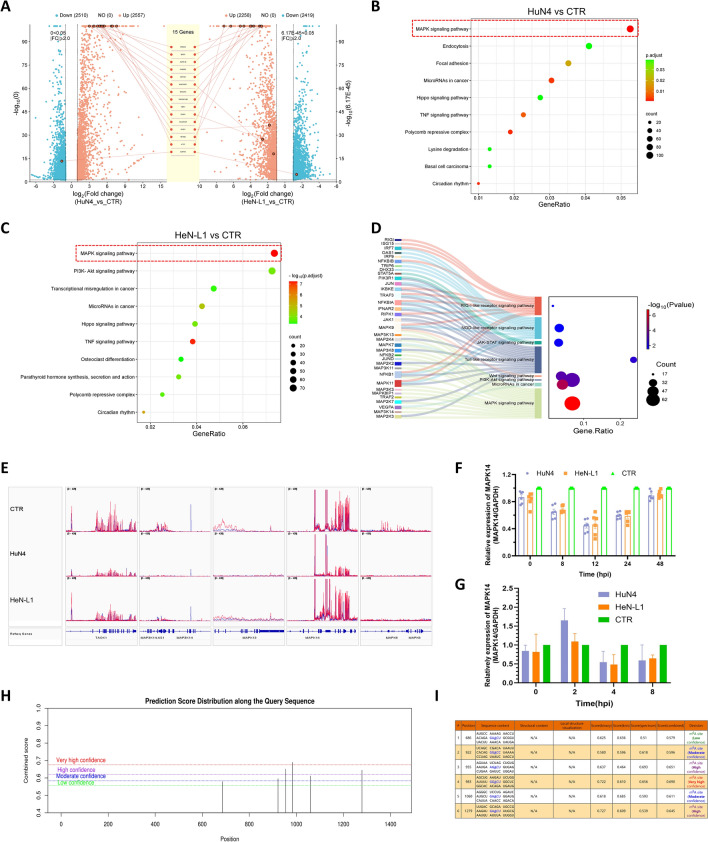
**Altered m**^6^**A landscape in the transcriptome of PRRSV-infected cells.** m^6^A-seq was used to detect the m^6^A modification levels in the mRNAs of PRRSV-infected PAMs. **A** Volcano maps illustrating the genes with different m^6^A modification levels between HuN4-infected PAMs (left lane) and HeN-L1-infected PAMs (right lane). **B** and **C** Using the KEGG database, cluster analysis was performed on genes with differential m^6^A modification levels. Bubble charts showing the clustering results of differentially expressed genes between HuN4-infected and uninfected cells (**B**) or HeN-L1-infected and uninfected cells **C**. The figure displays only the ten pathways with the smallest *p*-adjusted values (*p*-adjv). **D** Bubble charts were generated to display the genes with more than three m^6^A peaks and the specific signalling pathways to which they belong. The figure displays only the seven pathways with the smallest *p*-adjusted values (*p*-adjv). **E** Profiling of the m^6^A peaks in genes belonging to the MAPK signalling pathway via IGV software. The red lines indicate the m^6^A peaks, and the blue lines indicate the input signals. **F** and **G** RT‒qPCR was used to detect the expression level and stability of MAPK14 mRNA in HuN4- and HeN-L1-infected cells. **H** and **I** The online tool SRAMP was used to predict the m^6^A modification site in MAPK14 mRNA.

Given the profound impact of PRRSV infection on gene expression within host cells, we further used MeRIP-seq to investigate changes in m^6^A modification levels across the transcriptome of PAM cells after PRRSV infection. As shown in Figure 2A, approximately 27 329 m^6^A peaks, among the 4677 transcripts, exhibited significant differences between HeN-L1-infected cells and uninfected cells, with m^6^A levels significantly increased in 2258 genes and decreased in 2419 genes. Similarly, in HuN4-infected cells, 25 641 m^6^A peaks presented significant changes, with m^6^A levels upregulated in 2557 genes and downregulated in 2510 genes. Profiling the distribution of m^6^A peaks across the full length of mRNA revealed that, similar to those in uninfected cells, m^6^A modifications in virus-infected PAM cells predominantly appeared in coding regions (Additional file 2), indicating that m^6^A may be indirectly involved in regulating mRNA expression after viral infection. Mapping the reads to the porcine genome using IGV software revealed that genes with differential m^6^A levels were distributed among multiple cellular signalling pathways, including the PI3K-AKT pathway and cell cycle regulation (Additional file 2). In addition to mRNAs, m^6^A modifications have also been detected in noncoding RNAs, where miRNAs, in particular, play a key role in gene expression. Therefore, we conducted motif prediction analysis for differentially modified m^6^A peaks and identified several potential miRNAs. For example, miR-181c has been reported to counteract the PRRSV ORF4 gene to hinder viral replication, while miR-505 regulates viral infection by interacting with the PRRSV ORF7 gene. These findings suggest that m^6^A may serve as a regulatory mechanism for host miRNA expression in the context of PRRSV infection (Additional file 2).

To further explore how genes with differential m^6^A levels affect antiviral responses, we conducted gene cluster analysis. A heatmap (Additional file 3) illustrated differential gene expression patterns across samples, with distinct expression profiles in cells infected with the epidemic strain compared with those infected with the highly pathogenic strain. For example, the m^6^A modification levels of genes such as TRIM16 and MAP3K8 increased in cells infected with HeN-L1 but decreased in cells infected with HuN4, whereas the m^6^A levels of genes such as NOD2 and SOX9 decreased in HeN-L1-infected cells but increased in HuN4-infected cells. The GO and KEGG databases were used to analyse the signalling pathways associated with genes with altered m^6^A modification levels between HeN-L1- or HuN4-infected and uninfected cells. In the GO analysis (Additional file 3), genes with upregulated m^6^A peaks were enriched primarily in pathways such as negative regulation of DNA-templated transcription, negative regulation of the RNA biosynthetic process, and negative regulation of transcription by RNA polymerase II. While the results revealed m^6^A level changes in genes involved in RNA replication and cellular stress processes, further analysis is necessary to clarify the correlation between changes in m^6^A levels and specific signalling pathways. In the KEGG analysis (Figures 2B and C), we observed significant enrichment of MAPK signalling pathways, as well as the PI3K-AKT, Hippo, and TNF signalling pathways, suggesting that m^6^A modification may be crucial in modulating interactions between PRRSV and host cells. Interestingly, we screened genes with more than three m^6^A peaks and analysed the signalling pathways to which they belong. Pattern recognition receptor (PRR) signalling pathways associated with antiviral innate immune responses, such as the RIG-I-like, NOD-like, and Toll-like receptor signalling pathways, were enriched (Figure 2D). Previous studies have suggested that m^6^A modification plays a key role in antiviral innate immune responses by influencing the recognition of viral-derived nucleic acids by cytoplasmic PRRs. Our findings indicate that this mechanism may also play a significant role in PRRSV-infected PAMs.

### m^6^A modifications regulate the p38/MAPK signalling pathway during PRRSV infection

Among the diverse cellular signalling cascades, the p38 mitogen-activated protein kinase (MAPK) (p38/MAPK) signalling pathway plays a particularly pivotal role because it functions as a central metabolic hub that responds to various external and internal stress stimuli during PRRSV infection [23, 24]. This pathway is activated by a wide range of extracellular signals, such as proinflammatory cytokines, UV radiation, oxidative stress, and viral pathogens. Upon activation, p38/MAPK undergoes phosphorylation, which subsequently triggers a cascade of downstream signalling events that regulate cellular processes such as gene expression, apoptosis, differentiation, and cell cycle progression. Notably, we observed significant m^6^A enrichment in transcripts associated with the p38/MAPK signalling pathway during infection. These findings were identified through m^6^A-specific meRIP-seq analysis, followed by differential m^6^A peak comparisons between the infected and control groups. MAPK-related signalling pathways were significantly enriched in both the GO and KEGG analyses in the present study, with the MAPK9, MAPK11, and MAPK14 genes exhibiting multiple distinct m^6^A peaks. Mapping the m^6^A reads to the genome revealed that MAPK14 exhibited significantly different levels of m^6^A modification in both the HuN4 and CTR comparisons and the HeN-L1 and CTR comparisons, which indicated that MAPK14 may serve as a potential critical target for m^6^A-mediated regulation of PRRSV infection (Figure 2E).

To further investigate whether altered MAPK14 expression patterns are correlated with changes in m^6^A modification levels, we first examined the dynamic changes in MAPK14 mRNA levels in PRRSV-infected PAM cells. Compared with the uninfected cells, both HuN4 and HeN-L1 infection in the early phase (0–24 h) led to a decrease in MAPK14 mRNA levels (Figure 2F). However, from 24 to 48 h postinfection, MAPK14 mRNA levels partially recovered, which was consistent with the trend of protein expression. These findings indicate that MAPK14 transcriptional levels are affected during the early stages of PRRSV infection. m^6^A modification may enhance cytoplasmic mRNA decay, therefore affecting mRNA stability. To examine whether the observed decrease in MAPK14 mRNA is associated with reduced mRNA stability due to m^6^A modification, we used actinomycin D (actD) to inhibit intracellular mRNA transcription. The results (Figure 2G) revealed fewer MAPK14 mRNAs in PRRSV-infected cells than in uninfected cells, indicating the decreased stability of MAPK14 mRNA. Therefore, we preliminarily hypothesized that m^6^A modification regulates MAPK14 expression by influencing its mRNA stability. To further confirm the m^6^A modification sites within MAPK14 mRNA, we used the online prediction tool SRAMP to identify potential m^6^A modification sites. The results suggest that multiple modification sites exist in the MAPK14 gene, including three sites with high or very high confidence (Figures 2H and I). These findings provide a crucial foundation for further studies on how m^6^A modification regulates MAPK14 expression following PRRSV infection. In summary, our study indicates that the MAPK signalling pathway, particularly the MAPK14 gene, may be a key target through which m^6^A modification regulates the interaction between PRRSV and host cells.

## Discussion

The involvement of m^6^A in the viral genome has broader implications for our understanding of virus evolution and pathogenicity. The ability of viruses to exploit host mechanisms is key to their pathogenic success. As m^6^A is a type of epigenetic modification that can be manipulated by both the host and the virus, studying its role in viral infections may reveal new insights into viral evolution. In our study, seven m^6^A-enriched regions within the PRRSV genome were detected. Like those of other RNA viruses [25, 26], the m^6^A peaks are distributed across whole-genome RNA, which may indicate a general role for m^6^A in regulating PRRSV genomic RNA metabolism. Notably, the Nsp2 gene, which is the most variable gene of PRRSV [27, 28], contained the highest m^6^A peak, spanning approximately 178 nucleotides. The Nsp2 protein is a key viral nonstructural protein involved in PRRSV genomic RNA replication, viral polyprotein processing, and immune evasion. The presence of the highest m^6^A peak in the Nsp2 coding region suggests that this region is a potential target of m^6^A modification in the regulation of viral RNA synthesis. Given that the Nsp2 protein is also involved in modulating host immune responses and contributes to PRRSV pathogenesis, m^6^A modification in this region may also impact viral immune evasion strategies by regulating Nsp2 protein expression. Therefore, m^6^A modification may also serve as a strategy for PRRSV evolution.

In our study, by manipulating the expression of m^6^A enzymes, we found a positive relationship between m^6^A levels and PRRSV replication. As expected, further investigation revealed that treatment with 3-DAA significantly suppressed the replication of PRRSV. As shown in Additional file 2, 3-DAA inhibited virus replication from 20 µM to 25 µM but not from 5 µM to 10 µM at 48 hpi. Furthermore, the mRNA levels of N, GP5 and Nsp9 also decreased after 3-DAA treatment (Additional file 2), indicating that the methylation inhibitor may dampen virus replication via interference with viral mRNA transcription. Notably, such methylation inhibitors may serve as novel antiviral reagents in the context of acute PRRSV infection. Understanding how different viruses utilize m^6^A could help in the development of novel antiviral therapies that target these interactions. Emerging evidence highlights that the expression, localization, and functional dynamics of the host m^6^A machinery (writers, erasers, and readers) vary across species, which may shape diverse viral‒host interactions. For example, murine models may incompletely recapitulate human m^6^A-dependent viral replication owing to differences in METTL3 substrate specificity. This could explain why the Zika virus shows reduced m^6^A-dependent neurotropism in nonhuman primates than in humans. Additionally, variations in host cell types and immune responses may contribute to distinct m^6^A landscapes across different species. Viruses may evolve m^6^A as the optimized advantage for their primary hosts. For example, HIV-1 is enriched with m^6^A in regions critical for nuclear export in human T cells, whereas simian immunodeficiency virus (SIV) lacks analogous modifications, suggesting host-specific adaptation. Furthermore, species-specific m^6^A readers (e.g., YTHDF1/2/3) differentially regulate antiviral IFN responses. Compared with human coronavirus (HCoV-OC43), mouse hepatitis virus (MHV) replication is more sensitive to m^6^A depletion in murine cells, likely due to divergent YTHDF paralogue functions. Given that m^6^A modifications are widely utilized by various RNA viruses, including coronaviruses, flaviviruses, and paramyxoviruses, targeting m^6^A pathways could be a promising broad-spectrum antiviral strategy. For example, blocking METTL3 (e.g., with STM2457) reduces the replication of m^6^A-dependent viruses such as HIV-1 and SARS-CoV-2. Conversely, FTO inhibitors (e.g., FB23-2) enhance viral RNA sensing by stabilizing m^6^A-modified immunostimulatory RNAs, as shown in dengue and hepatitis C virus models. Moreover, CRISPR/Cas-based epitranscriptomic editing and m^6^A-regulated cis-elements in viral genomic RNAs (e.g., frameshift regions, IRESs) may further emphasize the potential applicability of m^6^A-based antiviral strategies to PRRSV and other pathogenic RNA viruses. Such therapies could disrupt the ability of viruses to exploit m^6^A modifications, potentially reducing their virulence and enhancing host defenses.

The p38/MAPK signalling pathway plays a pivotal role in the cellular response to various stresses, including viral infections. p38/MAPK is also critical in modulating the host immune response to viral infections. It activates transcription factors such as AP-1 and NF-κB, which are involved in the production of proinflammatory cytokines and interferons. The p38 MAPK pathway is involved in determining cell fate following viral infection. In response to viral infection, p38/MAPK can trigger apoptotic signalling pathways, which may limit viral replication by inducing cell death. However, p38 MAPK may also support viral survival by modulating antiapoptotic proteins, thereby enabling the virus to evade host immune responses and sustain infection. Our analysis suggested that the p38/MAPK signalling pathway may be a preliminary target for m^6^A modification in the regulation of PRRSV-host cell interactions. The term “preliminary targets” was used because our results indicate a strong association but do not yet establish causality. Functional validation experiments, such as m^6^A site mutagenesis or knockdown studies, are needed to confirm the direct regulatory role of m^6^A modifications on MAPK14 and the p38/MAPK pathway. Furthermore, because its activation serves both antiviral and proviral roles depending on the context, further research may be conducted to explore the dual functions of m^6^A regulation of the p38/MAPK signalling pathways in PRRSV infections. Moreover, other host genes, such as TRIM29, may also be involved in m^6^A regulation during PRRSV infection. TRIM29 plays a pivotal role in regulating immune responses against DNA and RNA viruses by modulating type I interferon signalling pathways, PERK-mediated ER stress immune responses, and inflammasome activation [29, 30]. Notably, TRIM29 expression has been shown to be upregulated via YTHDF1-mediated m^6^A modification [31]. Given the importance of TRIM29 in antiviral immunity, it is plausible that m^6^A modifications could similarly regulate TRIM29 expression during PRRSV infection, potentially impacting viral replication and immune evasion. Future studies might explore whether m^6^A methylation of TRIM29-related transcripts and the activity of YTHDF1 influence PRRSV pathogenesis and host defense mechanisms.

In summary, the importance of m^6^A in viral infections cannot be overstated. This modification serves as a crucial player in regulating various aspects of the viral lifecycle, from RNA stability and translation to immune evasion. By studying the role of m^6^A in specific viruses, researchers can gain valuable insights into the mechanisms of viral pathogenesis and host–virus interactions. Furthermore, understanding these processes opens new avenues for therapeutic interventions aimed at viral diseases. As our knowledge of m^6^A continues to expand, it is essential to explore its potential as a target for antiviral strategies, paving the way for innovative approaches to combat viral infections.

## Supplementary Information

**Additional file 1**. **Supplemental Materials and methods.**

**Additional file 2**. **Altered m**^6^**A landscape in the transcriptome of PRRSV-infected cells. **(A) and (B) Workflow of m^6^A MeRIP-seq. (C) Mapping the m^6^A peaks to the whole transcriptome in PRRSV-infected PAMs. (D) and (E) Profiling of the m^6^A peaks in genes related to cell cycle regulation and the PI3K-Akt signalling pathway via IGV software. The red lines indicate the m^6^A peaks, and the blue lines indicate the input signals. (F) Motif prediction analysis of differentially modified m^6^A peaks and potential miRNAs in PRRSV-infected cells compared with uninfected cells. (G) 3-DAA (5 µM, 10 µM, 20 µM or 25 µM) was added to the PAM cells, which were then incubated for 24 hours before virus inoculation (MOI=0.5). Twenty-four hours after virus infection, the supernatants were collected, and the viral titre was determined via a TCID_50_ assay. (H) 3-DAA (25 µM) was added to the PAM cells, which were then incubated for 24 hours before virus inoculation (MOI=0.5). Twenty-four hours after virus infection, the cells were collected, and total RNA was extracted to determine the viral mRNA level via RT‒qPCR. The relative expression of viral mRNA was compared with that of GAPDH. Statistical relevance was determined with an unpaired Student’s *t*‐test, *** *p* < 0.001.

**Additional file 3.** **Gene cluster analysis of different m**^6^**A-modified genes in PRRSV-infected cells.** (A) Heatmap illustrating differential gene expression patterns across samples, with distinct expression profiles in cells infected with the epidemic strain compared with those infected with the highly pathogenic strain. (B) The GO database was used to analyse the signalling pathways associated with genes with altered m^6^A modification levels between HeN-L1- or HuN4-infected and uninfected cells. The figure displays only the nine pathways with the smallest *p*-adjusted values (*p*-adjv).

## Data Availability

The data that support the findings of this study are available from the corresponding author (JT) upon reasonable request.

## References

[1] Murakami S, Jaffrey SR (2022) Hidden codes in mRNA: control of gene expression by m^6^A. Mol Cell 82:2236–2251 35714585 10.1016/j.molcel.2022.05.029 PMC9216239

[2] Tong J, Zhang W, Chen Y, Yuan Q, Qin NN, Qu G (2022) The emerging role of RNA modifications in the regulation of antiviral innate immunity. Front Microbiol 13:845625 35185855 10.3389/fmicb.2022.845625 PMC8851159

[3] Yu PL, Cao SJ, Wu R, Zhao Q, Yan QG (2021) Regulatory effect of m^6^ A modification on different viruses. J Med Virol 93:6100–6115 34329499 10.1002/jmv.27246

[4] Jiang X, Liu B, Nie Z, Duan L, Xiong Q, Jin Z, Yang C, Chen Y (2021) The role of m^6^A modification in the biological functions and diseases. Signal Transduct Target Ther 6:74 33611339 10.1038/s41392-020-00450-x PMC7897327

[5] Sendinc E, Shi Y (2023) RNA m^6^A methylation across the transcriptome. Mol Cell 83:428–441 36736310 10.1016/j.molcel.2023.01.006

[6] Chen T, Greene GH, Motley J, Mwimba M, Luo GZ, Xu G, Karapetyan S, Xiang Y, Liu C, He C, Dong X (2024) m^6^A modification plays an integral role in mRNA stability and translation during pattern-triggered immunity. Proc Natl Acad Sci U S A 121:e2411100121 39116132 10.1073/pnas.2411100121 PMC11331096

[7] Dominissini D, Moshitch-Moshkovitz S, Schwartz S, Salmon-Divon M, Ungar L, Osenberg S, Cesarkas K, Jacob-Hirsch J, Amariglio N, Kupiec M, Sorek R, Rechavi G (2012) Topology of the human and mouse m^6^A RNA methylomes revealed by m^6^A-seq. Nature 485:201–206 22575960 10.1038/nature11112

[8] Huang S, Wylder AC, Pan T (2024) Simultaneous nanopore profiling of mRNA m^6^A and pseudouridine reveals translation coordination. Nat Biotechnol 42:1831–1835 38321115 10.1038/s41587-024-02135-0 PMC11300707

[9] Chen Y, Wang W, Zhang W, He M, Li Y, Qu G, Tong J (2023) Emerging roles of biological m(6)A proteins in regulating virus infection: a review. Int J Biol Macromol 253:126934 37722640 10.1016/j.ijbiomac.2023.126934

[10] Wang W, Jin Y, Xie Z, He M, Li J, Wang Z, Ma S, Zhang W, Tong J (2024) When animal viruses meet N^6^-methyladenosine (m^6^A) modifications: for better or worse? Vet Res 55:171 39695760 10.1186/s13567-024-01424-5 PMC11657938

[11] Ruedas-Torres I, Rodriguez-Gomez IM, Sanchez-Carvajal JM, Larenas-Munoz F, Pallares FJ, Carrasco L, Gomez-Laguna J (2021) The jigsaw of PRRSV virulence. Vet Microbiol 260:109168 34246042 10.1016/j.vetmic.2021.109168

[12] Wensvoort G, Terpstra C, Pol JM, ter Laak EA, Bloemraad M, de Kluyver EP, Kragten C, van Buiten L, den Besten A, Wagenaar F, Broekhuijsen JM, Moonen PLJM, Zetstra T, de Boer EA, Tibben HJ, de Jong MF, van ‘t Veld P, Greenland GJR, van Gennep JA, Voets MTh, Verheijden JHM, Braamskamp J (1991) Mystery swine disease in The Netherlands: the isolation of Lelystad virus. Vet Q 13:121–130 1835211 10.1080/01652176.1991.9694296

[13] An TQ, Tian ZJ, Xiao Y, Li R, Peng JM, Wei TC, Zhang Y, Zhou YJ, Tong GZ (2010) Origin of highly pathogenic porcine reproductive and respiratory syndrome virus, China. Emerg Infect Dis 16:365–367 20113592 10.3201/eid1602.090005 PMC2957991

[14] Li Y, Wang X, Bo K, Wang X, Tang B, Yang B, Jiang W, Jiang P (2007) Emergence of a highly pathogenic porcine reproductive and respiratory syndrome virus in the Mid-Eastern region of China. Vet J 174:577–584 17869553 10.1016/j.tvjl.2007.07.032

[15] Tong GZ, Zhou YJ, Hao XF, Tian ZJ, An TQ, Qiu HJ (2007) Highly pathogenic porcine reproductive and respiratory syndrome, China. Emerg Infect Dis 13:1434–1436 18252136 10.3201/eid1309.070399 PMC2857295

[16] Zhou L, Wang Z, Ding Y, Ge X, Guo X, Yang H (2015) NADC30-like strain of porcine reproductive and respiratory syndrome virus, China. Emerg Infect Dis 21:2256–2257 26584305 10.3201/eid2112.150360 PMC4672414

[17] Zhang H, Leng C, Ding Y, Zhai H, Li Z, Xiang L, Zhang W, Liu C, Li M, Chen J, Bai Y, Kan Y, Yao L, Peng J, Wang Q, Tang YD, An T, Cai X, Tian Z, Tong G (2019) Characterization of newly emerged NADC30-like strains of porcine reproductive and respiratory syndrome virus in China. Arch Virol 164:401–411 30353281 10.1007/s00705-018-4080-7

[18] Lin C, Zeng M, Song J, Li H, Feng Z, Li K, Pei Y (2023) PRRSV alters m^6^A methylation and alternative splicing to regulate immune, extracellular matrix-associated function. Int J Biol Macromol 253:126741 37696370 10.1016/j.ijbiomac.2023.126741

[19] Gong X, Liang Y, Wang J, Pang Y, Wang F, Chen X, Zhang Q, Song C, Wang Y, Zhang C, Fang X, Chen X (2024) Highly pathogenic PRRSV upregulates IL-13 production through nonstructural protein 9-mediated inhibition of N6-methyladenosine demethylase FTO. J Biol Chem 300:107199 38508309 10.1016/j.jbc.2024.107199 PMC11017062

[20] Liu J, Yue Y, Han D, Wang X, Fu Y, Zhang L, Jia G, Yu M, Lu Z, Deng X, Dai Q, Chen W, He C (2014) A METTL3-METTL14 complex mediates mammalian nuclear RNA N^6^-adenosine methylation. Nat Chem Biol 10:93–95 24316715 10.1038/nchembio.1432 PMC3911877

[21] Roundtree IA, Evans ME, Pan T, He C (2017) Dynamic RNA modifications in gene expression regulation. Cell 169:1187–1200 28622506 10.1016/j.cell.2017.05.045 PMC5657247

[22] Chen LJ, Liu HY, Xiao ZY, Qiu T, Zhang D, Zhang LJ, Han FY, Chen GJ, Xu XM, Zhu JH, Ding YQ, Wang SY, Ye YP, Jiao HL (2023) IGF2BP3 promotes the progression of colorectal cancer and mediates cetuximab resistance by stabilizing EGFR mRNA in an m^6^A-dependent manner. Cell Death Dis 14:581 37658049 10.1038/s41419-023-06099-y PMC10474290

[23] Chen D, Xu S, Jiang R, Guo Y, Yang X, Zhang Y, Zhou L, Ge X, Han J, Guo X, Yang H (2022) IL-1beta induced by PRRSV co-infection inhibited CSFV C-strain proliferation via the TLR4/NF-kappaB/MAPK pathways and the NLRP3 inflammasome. Vet Microbiol 273:109513 35952491 10.1016/j.vetmic.2022.109513

[24] Hou J, Wang L, Quan R, Fu Y, Zhang H, Feng WH (2012) Induction of interleukin-10 is dependent on p38 mitogen-activated protein kinase pathway in macrophages infected with porcine reproductive and respiratory syndrome virus. Virol J 9:165 22909062 10.1186/1743-422X-9-165 PMC3441385

[25] Kennedy EM, Bogerd HP, Kornepati AV, Kang D, Ghoshal D, Marshall JB, Poling BC, Tsai K, Gokhale NS, Horner SM, Cullen BR (2016) Posttranscriptional m^6^A editing of HIV-1 mRNAs enhances viral gene expression. Cell Host Microbe 19:675–685 27117054 10.1016/j.chom.2016.04.002 PMC4867121

[26] Mishra T, Phillips S, Zhao Y, Wilms B, He C, Wu L (2024) Epitranscriptomic m^6^A modifications during reactivation of HIV-1 latency in CD4^+^ T cells. MBio 15:e0221424 39373537 10.1128/mbio.02214-24 PMC11559067

[27] Bai YZ, Wang S, Sun Y, Liu YG, Zhang HL, Wang Q, Huang R, Rao CH, Xu SJ, Tian ZJ, An TQ, Cai XH, Tang YD (2024) The full-length nsp2 replicase contributes to viral assembly in highly pathogenic PRRSV-2. J Virol 99:e0182124 39601570 10.1128/jvi.01821-24 PMC11784222

[28] Cao S, Liu J, Ding G, Shao Q, Wang B, Li Y, Feng J, Zhao Y, Liu S, Xiao Y (2020) The tail domain of PRRSV NSP2 plays a key role in aggrephagy by interacting with 14-3-3epsilon. Vet Res 51:104 32811532 10.1186/s13567-020-00816-7 PMC7433210

[29] Wang J, Lu W, Zhang J, Du Y, Fang M, Zhang A, Sungcad G, Chon S, Xing J (2024) Loss of TRIM29 mitigates viral myocarditis by attenuating PERK-driven ER stress response in male mice. Nat Commun 15:3481 38664417 10.1038/s41467-024-44745-x PMC11045800

[30] Xing J, Zhang A, Zhang H, Wang J, Li XC, Zeng MS, Zhang Z (2017) TRIM29 promotes DNA virus infections by inhibiting innate immune response. Nat Commun 8:945 29038422 10.1038/s41467-017-00101-w PMC5643338

[31] Hao L, Wang JM, Liu BQ, Yan J, Li C, Jiang JY, Zhao FY, Qiao HY, Wang HQ (2021) m^6^A-YTHDF1-mediated TRIM29 upregulation facilitates the stem cell-like phenotype of cisplatin-resistant ovarian cancer cells. Biochim Biophys Acta Mol Cell Res 1868:118878 33011193 10.1016/j.bbamcr.2020.118878

