# Supplementary material for: Dynamic interplay between RNA N6-methyladenosine modification and porcine reproductive and respiratory syndrome virus infection
Source: Vet Res. 2025 Mar 22;56:64. doi: 10.1186/s13567-025-01495-y (PMC11929310; doi:10.1186/s13567-025-01495-y)
Supplement: Supplementary file 1 — Additional file 1. Supplemental Materials and methods. [file 13567_2025_1495_MOESM1_ESM.docx]

**Additional file 1 supplemental Materials and methods**

**Cells and viruses**

Porcine alveolar macrophages (PAMs) were isolated from 4-8 weeks-old SPF piglets and cultured in RPMI-1640 medium (Procell Bio China, #PM150110), containing 10% fetal bovine serum (FBS, Sigma-Aldrich, #F8318,) and 1% penicillin/streptomycin (P/S, Thermo Fisher Scientific, #15140122) at 37 °C with 5% CO_2_.

The PRRSV HuN4 strain or the PRRSV NADC30-like strain were subjected to the PAMs using a multiplicity of infection (MOI) of 0.1. Virus was inoculated with the cells for 1 h at 37 °C in a humidified atmosphere with 5% CO_2_ and the virus containing-supernatants were replaced by RPMI-1640 supplemented with 2%FBS until the required time point. Viral stocks for both strains were titrated and prepared in parallel to ensure comparable infectivity. All experiments were performed in triplicate for three times.

**Viral RNA pretreatment for Me-RIP seq**

Viral RNA was extracted using TRIzol reagent (Thermo-fisher Scientific, #15596018CN) and subsequently fragmented into lengths of 80-200 nt by heating in fragmentation buffer (10 mM Tris-HCl, pH 7.0, 10 mM ZnCl₂) at 94 °C for 5 min, followed by immediate cooling on ice to halt the reaction.

**Me-RIP seq analysis**

MeRIP-seq was performed by DIATRE Biotechnology (Shanghai, China). For immunoprecipitation, 10 µg of anti-m6A antibody (Abcam, #ab208577,) was used per reaction. RNA libraries were prepared using the NEBNext Ultra II Directional RNA Library Prep Kit (New England Biolabs, #E7760S) according to the manufacturer’s protocol. RNA fragmentation was achieved as described, and the size selection of ~200 nt fragments was performed using AMPure XP beads. Sequencing was carried out on an Illumina NovaSeq platform to generate 150 bp paired-end reads.

The meRIP-seq data is analyzed as follows: Fastqc evaluation of the quality of the original data; BWA software was used to map reads against the reference genome; Aligned SAM files were sorted by Samtools; Signal distribution (peak calling) was identified by MACS2; Peak and motif annotation results were generated by bedtools and HOMER respectively; Statistical analysis and function enrichment analysis were completed using corresponding R packages.
