# Supplementary figures and images for: Dynamic interplay between RNA N6-methyladenosine modification and porcine reproductive and respiratory syndrome virus infection
Source: Vet Res. 2025 Mar 22;56:64. doi: 10.1186/s13567-025-01495-y (PMC11929310; doi:10.1186/s13567-025-01495-y)

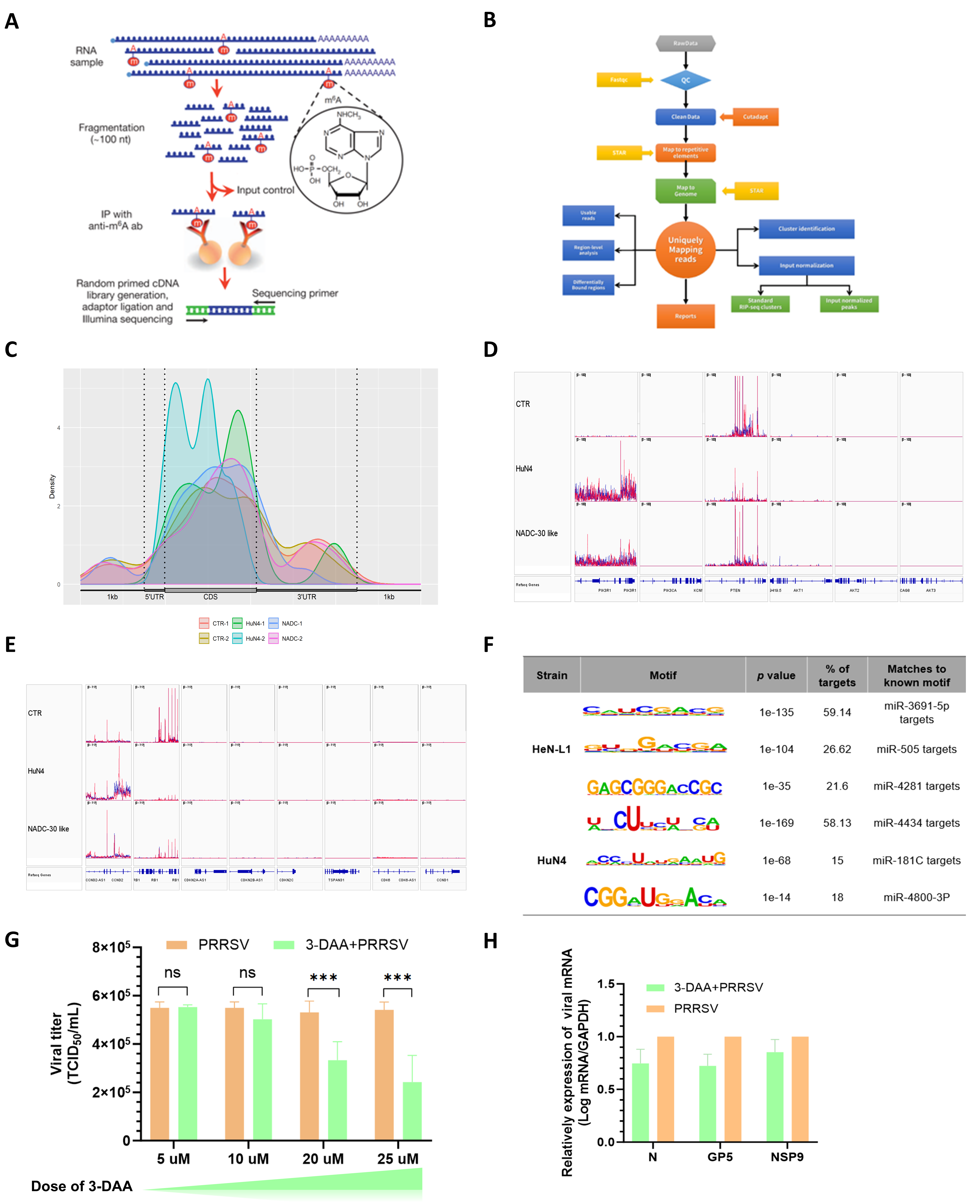

Supplement: Supplementary file 2 — Additional file 2. Altered m6A landscape in the transcriptome of PRRSV-infected cells. (A) and (B) Workflow of m6A MeRIP-seq. (C) Mapping the m6A peaks to the whole transcriptome in PRRSV-infected PAMs. (D) and (E) Profiling of the m6A peaks in genes related to cell cycle regulation and the PI3K-Akt signalling pathway via IGV software. The red lines indicate the m6A peaks, and the blue lines indicate the input signals. (F) Motif prediction analysis of differentially modified m6A peaks and potential miRNAs in PRRSV-infected cells compared with uninfected cells. (G) 3-DAA (5 µM, 10 µM, 20 µM or 25 µM) was added to the PAM cells, which were then incubated for 24 hours before virus inoculation (MOI=0.5). Twenty-four hours after virus infection, the supernatants were collected, and the viral titre was determined via a TCID50 assay. (H) 3-DAA (25 µM) was added to the PAM cells, which were then incubated for 24 hours before virus inoculation (MOI=0.5). Twenty-four hours after virus infection, the cells were collected, and total RNA was extracted to determine the viral mRNA level via RT‒qPCR. The relative expression of viral mRNA was compared with that of GAPDH. Statistical relevance was determined with an unpaired Student’s t‐test, *** p < 0.001. [file 13567_2025_1495_MOESM2_ESM.tif]

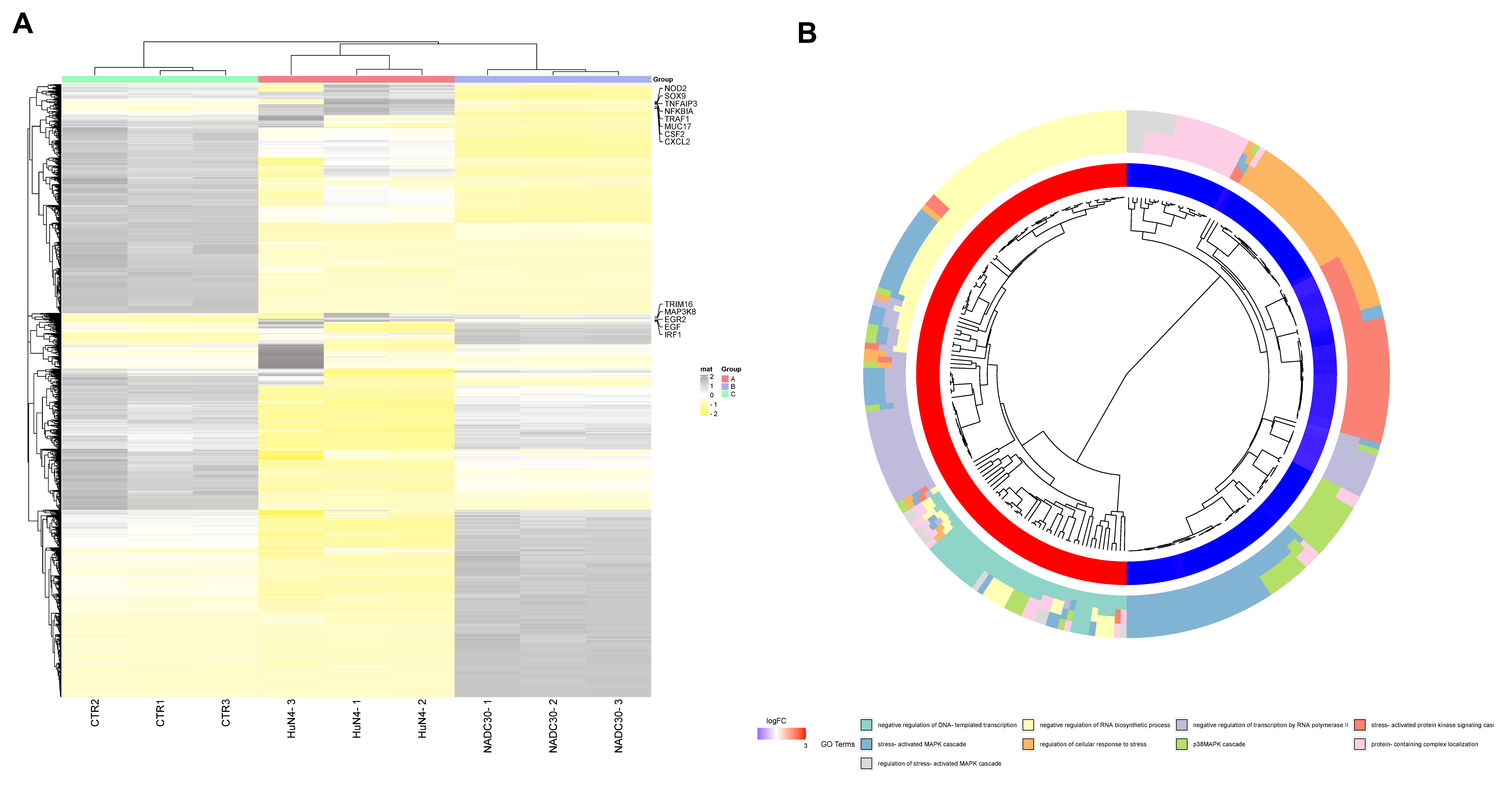

Supplement: Supplementary file 3 — Additional file 3. Gene cluster analysis of different m6A-modified genes in PRRSV-infected cells. (A) Heatmap illustrating differential gene expression patterns across samples, with distinct expression profiles in cells infected with the epidemic strain compared with those infected with the highly pathogenic strain. (B) The GO database was used to analyse the signalling pathways associated with genes with altered m6A modification levels between HeN-L1- or HuN4-infected and uninfected cells. The figure displays only the nine pathways with the smallest p-adjusted values (p-adjv). [file 13567_2025_1495_MOESM3_ESM.tif]
